# Modeling and mitigating supply chain disruptions as a bilevel network flow problem

**DOI:** 10.1007/s10287-022-00421-3

**Published:** 2022-02-28

**Authors:** René Y. Glogg, Anna Timonina-Farkas, Ralf W. Seifert

**Affiliations:** 1 École Polytechnique Fédérale de Lausanne (EPFL), EPFL-CDM-MTEI-TOM, ODY 1.03, Station 5, 1015 Lausanne, Switzerland; 2 International Institute for Management Development (IMD), Chemin de Bellerive 23, 1003 Lausanne, Switzerland

**Keywords:** Supply chain management, Supply chain resilience, Risk mitigation, Stochastic bilevel optimization, Benders decomposition

## Abstract

Years of globalization, outsourcing and cost cutting have increased supply chain vulnerability calling for more effective risk mitigation strategies. In our research, we analyze supply chain disruptions in a production setting. Using a bilevel optimization framework, we minimize the total production cost for a manufacturer interested in finding optimal disruption mitigation strategies. The problem constitutes a convex network flow program under a chance constraint bounding the manufacturer’s regrets in disrupted scenarios. Thus, in contrast to standard bilevel optimization schemes with two decision-makers, a leader and a follower, our model searches for the optimal production plan of a manufacturer in view of a reduction in the sequence of his own scenario-specific regrets. Defined as the difference in costs of a *reactive plan*, which considers the disruption as unknown until it occurs, and a benchmark *anticipative plan*, which predicts the disruption in the beginning of the planning horizon, the regrets allow measurement of the impact of scenario-specific production strategies on the manufacturer’s total cost. For an efficient solution of the problem, we employ generalized Benders decomposition and develop customized feasibility cuts. In the managerial section, we discuss the implications for the risk-adjusted production and observe that the regrets of long disruptions are reduced in our mitigation strategy at the cost of shorter disruptions, whose regrets typically stay far below the risk threshold. This allows a decrease of the production cost under rare but high-impact disruption scenarios.

## Introduction

Supply chains play a vital role in the global economy. This has been demonstrated in various industry studies, e.g., by Klaus Schwab in the World Economic Forum (2012, 2013) and by van Opstal (2009). According to Christopher and Lee (2004) and Christopher and Peck (2004), the vulnerability of supply chains towards external disturbances is increasing over time.

The results of a survey by The Business Continuity Institute (2012) suggest that “73% of global supply chains are facing at least one major disruption over a period of one year.” For instance, the COVID-19 pandemic has imposed a burden of an unprecedented scale on the supply chain resilience of a variety of industries, including the pharmaceutical industry, whose functioning is crucial because of the importance of matching supply and demand for life-saving medicine. Another example is the Japanese company Hitachi Chemical, whose market share is over 90% for the production of a specific resin for microchips. The company is exposed to the risk of natural disasters such as earthquakes, tsunamis, nuclear incidents and power shortages. A disruption in the company’s production would affect manufacturing of various IT-related devices worldwide, as argued in The Economist (2011). Besides these examples, high commodity price volatility has drawn practitioners’ attention to the cost of vulnerability in supply chains. In general, although the primary strategy of profit-oriented manufacturers remains in scenario-specific production optimization, the models aiming for resilience towards disruptions at a reasonable cost are in great demand. The decision-makers facing disruption risk are interested in adopting the best possible mitigation strategy taking uncertainties explicitly into account. This need has only intensified with the advent of the COVID-19 pandemic, which, as it has been observed, may cause various different types of disruptions effecting supply chains at multiple levels and occurring simultaneously (Sodhi and Tang 2020). The models capable of accounting for this variety are on the forefront of supply chain management under uncertainty.

The main contribution of our research is in the development of a general model which provides considerable flexibility in the type of disruptions it incorporates and which allows to account for the difference in strategies the manufacturer adopts in view of a disruption. Specifically, we develop a finite time horizon bilevel stochastic optimization model of a supply chain network with production disruptions that are uncertain in both time and severity. The finite horizon assumption results in a model which is adequate in addressing questions arising during limited product life cycles. It is also straightforward to extend the model to the case with a rolling horizon in order to account for multiple decisions to be taken over a longer time period. Overall, we formulate the problem as a convex non-differentiable minimum-cost network flow program accounting for a wide range of possible cost and disruption structures such as those arising due to loading and raw material switching effects. To account for the difference in strategies to deal with disruptions, we minimize the total production cost introducing a chance constraint which bounds the surplus in a manufacturer’s regret following from the non-anticipative strategy of each disruption scenario. The evaluation of these regrets requires a solution of a sequence of problems depending on the manufacturer’s optimal network flow.

Modeling uncertainty in production, we build a set of possible disruption scenarios and characterize the loss the decision-maker observes in each scenario realization by the means of a *regret function*. The goal is to find a production schedule such that the value of this regret function does not exceed some threshold with high probability, where the threshold, the exceedance probability and the capacitated cost function form are assumed by the decision-maker. The regret function is defined as the distance between optimal values of a *reactive* and an *anticipative* optimization problem. The *reactive problem* minimizes the production cost naturally, allowing the disruption to be unknown until it occurs. In turn, the *anticipative problem* plays the role of an idealistic benchmark strategy of a manufacturer, considering the disruption scenario as defined with certainty at the beginning of the planning horizon. Thus, our problem exhibits bilevel structure as some constraints are defined through additional optimization problems (Dempe 2002). Specifically, our setup allows us to compare the impact of deciding to wait for the disruption as opposed to preparing for disruptions ahead of time.

Bilevel optimization problems are commonly studied in supply chain management in the context of optimization schemes with two decision-makers, a leader and a follower, see, e.g., Yue and You (2017), as well as in the context of decentralized supply chains, see, e.g., Haque et al. (2020) or Hsueh (2015). In contrast, our model searches for the optimal production plan of a manufacturer in view of a reduction in the sequence of his own regrets. The problem of optimization of regrets (i.e., lower-level problem) is embedded within the manufacturer’s cost minimization (i.e., the upper-level problem) via the use of chance constraints. The use of regret functions is well established in decision theory (Loomes and Sugden 1982; Bell 1982). However, a chance constraint formulation dependent on the evaluation of optimal regrets makes the problem difficult to solve efficiently. Our solution method is adapted to the problem structure discussed in this article.

In the numerical section, we apply a generalized Benders decomposition inspired by the approach outlined in Zheng et al. (2015) and first introduced in Geoffrion (1972). We customize the procedure by introducing efficient feasibility cuts to the optimization problem without chance constraints. For this, we demonstrate that the Benders feasibility problem corresponds to the convex minimum-cost network flow problem. For its solution, we compute theoretically optimal dual multipliers corresponding to flow equilibrium constraints and use them to construct feasibility cuts as described by Fischetti et al. (2016) and Geoffrion (1972). Our approach allows for efficiency in high dimensions with a large number of disruption scenarios.

The model we present has a flexible structure for the generation of disruption scenarios. It depends on the disruption starting point, its length and severity. Specifically, a disruption in our model is described by a change in a set of cost functions for some production period. Due to this, we account both for the disruptions representing the total shutdown of a production (e.g., due to unprecedented events such as COVID-19) and the disruptions in which some partial capacity remains available during the disrupted period (e.g., due to flood events in a single production site). Therefore, the model is able to compare mitigation strategies observed in practice: for example, in case a company facing a disruption risk decides between carrying additional inventory to diminish losses when the disruption occurs (*risk mitigation inventory*) and reserving some capacity from an alternative source unaffected by the disruption at a potentially higher cost (*agility capacity*).

In the managerial part of the article, we perform numerical experiments in which the inventories to be carried are determined under various combinations of input parameters. For simplicity, we assume the demand to be deterministic in our article and test various demand patterns. By this, we suppose that the constant trend corresponds to the maturity stage of the product life cycle where the demand can be described by a stationary process, as argued by Lücker and Seifert (2017). Clearly, linearly increasing/decreasing and bell-shape demands correspond to other periods of the product life cycle. Note that an extension of the model would be applicable for uncertain demands including non-stationary cases: one could quantize demand scenarios solving the expectation-minimization problem to account for the uncertainty in demand. In this article, we do not follow this path and focus instead on the impact of disruptions on a manufacturer’s optimal production and inventory quantities. In particular, we observe that the mitigation inventory tends to decrease for costly but extremely rare disruptions, which can be neglected due to the use of chance constraints. We refer to this behavior as *risk clustering* and demonstrate it in Sect. 7 of the article.

The article is structured as follows: Sect. 2 provides a literature review. Section 3 describes the model, while Sect. 4 focuses on the numerical method for the solution of the optimization problem via Benders decomposition. We describe our scenario generation method in Sect. 5 and perform functional analysis in Sect. 6. Managerial insights are presented in Sect. 7.

## Literature review

Practitioners are interested in discovering efficient ways to diminish the amount of risk that supply chains are exposed to. Nevertheless, doing business requires the acceptance of some level of risk within organizations (Olson and Wu 2011) and, thus, a supply chain can never be risk-free (Xie et al. 2011). Numerous articles analyze and classify potential supply chain risks, which is a difficult task due to the complexity of modern supply chains (Pfohl et al. 2011). Literature reviews on the topic of supply chain risk management can be found in Dong and Tomlin (2012) or Heckmann et al. (2015). In our research, we are interested in understanding risk related to supply chain disruptions, which Barroso et al. (2008) define as foreseeable or unforeseeable events directly affecting the usual operation and stability of an organization or a supply chain. Such disruptions can have enormous financial consequences. For instance, Hendricks and Singhal (2005) analyze a time span from one year prior to two years after a supply chain disruption and conclude that there is an average decrease in stock returns of nearly 40% due to the effect of such a disruption.

We contribute to this stream of literature by introducing an approach which models and measures the impact of production disruptions on supply chains taking strategy-specific regrets into account. This allows us to obtain and analyze the optimal risk mitigation, as well as to focus on plans such as holding risk mitigation inventory or leveraging agility capacity, whose importance is broadly recognized. For example, Lücker and Seifert (2017) determine optimal inventory levels under supply chain disruptions with deterministic demand for a pharmaceutical firm. Via this analysis, the authors compare the benefits of the aforementioned mitigation strategies. Their approach is extended to serial multi-echelon supply chains in Lücker et al. (2019). Furthermore, dual sourcing and mitigation inventory are examined in detail by Tomlin (2006). Also, additional mitigation strategies are reviewed in Chopra and Sodhi (2004) and in Snyder et al. (2016), as well as in Tang (2006) as part of a more general overview of supply chain risk management. Nevertheless, current literature has not yet accounted for the idealistic desire of manufacturers to prepare for particular disruptions only, as well as for their regrets in case of scenario-specific loss deviations. In our article, we address this issue using chance constraints.

Our article also goes in line with the stream of literature analyzing the behavior of disruptions within supply chains. Liberatore et al. (2012) assess disruption propagation in supply chains, while systemic propagation risks are considered in Scheibe and Blackhurst (2018). Simchi-Levi et al. (2018) study supply chain robustness according to multiple network topologies. Ivanov et al. (2017) review the literature on disruption recovery, and Lim et al. (2013) show that higher expected costs occur when disruption probabilities are underestimated. Analogous to this result, in our research we observe that the expected loss of a decision-maker who actively invests in mitigation strategies is lower than that of a decision-maker who invests less actively.

In our work, we formulate a convex minimum-cost network flow problem in order to model supply chain disruptions. An introduction to the theory of such problems can be found in Bertsekas (1998). We define a measure of the loss incurred by a decision-maker whenever a disruption occurs in the form of a *regret function*. We introduce the regret into our model using a chance constraint formulation analogous to approaches bounding value-at-risk (the possibility of using VaR in multi-period inventory management problems is explored by Luciano et al. (2003). Stochastic optimization with chance constraints is successfully applied to solve a wide variety of problems (e.g., Zheng et al. 2015) and proves to be accurate and efficient for managing inventory and production in our study. Mathematically, the resulting problem exhibits bilevel structure, since the decision-maker is permitted to re-plan after a disruption occurs and as optimal decisions of upper- and lower-level problems influence one another. The opportunity to re-plan is quite natural when dealing with probability bounds and can also follow the structure discussed in Outrata (1988) via the use of combinatorial inequality constraints.

The use of bilevel optimization is also motivated in decentralized supply chains which often exhibit a nested structure of decision-making problems. This setting typically lends itself to game-theoretic considerations. Specifically, a commonly used approach is to model a decentralized supply chain as a Stackelberg-leader-follower game, which results in a bilevel programming problem in which the follower’s decision is being searched for in the lower-level optimization problem. A classic method to solve linear problems of this type uses genetic algorithms and is discussed in Calvete et al. (2008) with many extensions studied since. For example, in Yue and You (2017) the lower-level optimization problem contains discrete decisions, necessitating the development of a novel mixed integer bilevel programming modeling framework since standard methods do not apply. Another approach considers each individual non-cooperative entity in a supply chain as the decision-maker at a lower level, while an upper-level decision is taken by some independent entity accounting for the solutions at the lower level. Such a framework is discussed in Haque et al. (2020) or in Hsueh (2015).

Irrespective of an application area, the common approach to solve bilevel problems whenever the lower-level exhibits convex structure is to use Karush-Kuhn-Tucker conditions to replace the lower level problem with a particular set of constraints (Bard 2013). However, even under simplifying assumptions such as piece-wise linear convex costs, the complexity of the resulting problem grows quickly in the number of periods. This complexity stems primarily from the rapidly growing number of disruption scenarios which need to be considered. However, if scenarios are independent of each other the problem is suitable for decomposition approaches. We use generalized Benders decomposition (Geoffrion 1972) with customized feasibility cuts to obtain the solution efficiently. We decrease the number of computations using the regret bounding procedure which exploits the structure of the Benders subproblems. Importantly, our formulation does not require optimality cuts. Benders decomposition has also been applied in a two-stage context by Adulyasak et al. (2015), who solve a stochastic production routing problem with demand uncertainty. Unlike their problem, our approach is a direct consequence of the regret formulation able to separate between decisions before and after a disruption scenario is realized. Differently, Adulyasak et al. (2015) separate between tactical and operational decisions in routing. Additionally, we analyze the optimal solution from a managerial perspective to obtain insights into the structure of the optimal mitigation inventory and the trade-offs between mitigation strategies.

## Mathematical model

In this section, we firstly present a baseline convex minimum-cost network flow problem which is used to determine the optimal production schedule over a given time horizon with deterministic demands. Afterwards, we introduce an extension to this mathematical model incorporating disruption scenarios via a chance constraint and we discuss how to solve the resulting problem efficiently. Consider a manufacturer working under production and inventory capacity constraints. Constrained capacities occur whenever a real-world business has insufficient production or inventory to meet the demand. In particular, this can be due to equipment or production line limitations, poor reliability, planning constraints, utilities capacity such as available electric power, water etc. In this work, we denote production periods by $t\in \{1,\ldots ,T\}$ and the deterministic demand in each period by $d_t$. If production in period $t$ is disrupted, the production capacity drops. In order to minimize the production cost under capacity constraints and to account for possible disruptions, the manufacturer decides on the periodic production quantities $P_t,\; \forall t=1,\ldots ,T$ and the inventories $I_t,\; \forall t = 1,\ldots ,T-1$. In our setting, the manufacturer assumes zero initial inventory $I_0=0$ available in the beginning of period $t=1$. A non-zero inventory $I_0$ can be easily dealt with by reformulating the problem. For example, such an inventory can be thought of as a zero-cost production prior to period $t=1$ with fixed capacity bounds.

**Fig. 1:**
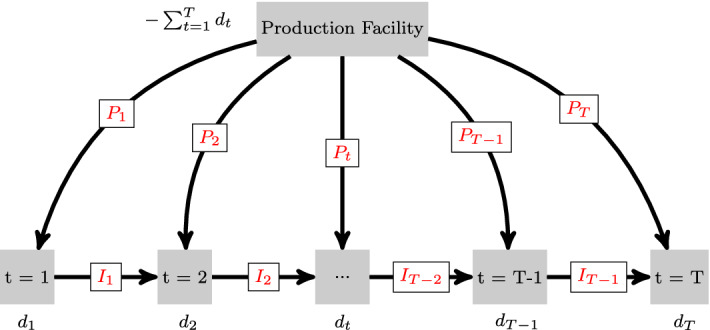
Undisrupted multi-period production scheme

To state the optimization problem in the network flow form, we define a *directed* graph $G=(V,A)$, where $V=\{0,1,\ldots ,T\}$ is the set of nodes and *A* is the set of arcs. We denote the production node by $0$ and let $A^\text {prod}=\big \{(0,1),\ldots ,(0,T)\big \}$ (resp. $A^\text {inv}=\big \{(1,2),\ldots ,(T-1,T)\big \}$) be the subset of production (resp. inventory) arcs. Thus, $A=A^\text {prod} \cup A^\text {inv}$ and $A^\text {prod} \cap A^\text {inv} = \emptyset$. The supply outgoing from node 0 is in equilibrium with the demand, i.e., $d_0+\sum _{t=1}^T d_t = 0$, where $d_0$ is the supply (or negative demand) at this node (Fig. 1). The cost functions are denoted by $C_{ij}(\cdot )$ for each arc $(i,j) \in A$, while $y_{ij}$ and $\kappa _{ij}$ represent the arc flows and the capacity constraints respectively. The flow in the network is denoted by $y = (y_{ij})_{(i,j) \in A}$ and the total cost function of the flow is $C(y) = \sum _{(i,j) \in A} C_{ij}(y_{ij})$.

Therefore, the convex minimum-cost network flow problem is formulated in the following form:

1 $$\begin{aligned} v= & {} \mathop {\min }\limits _{y}\quad {\sum _{(i,j) \in A} C_{ij}(y_{ij}) }\nonumber \\&\text {s.t}\quad \sum _{(j,i) \in A} y_{ji}^{} - \sum _{(i,j) \in A} y_{ij}^{}=d_i\; \forall \; i \in V\nonumber \\&\qquad y_{ij}\le \kappa _{ij},\; \forall (i,j) \in A,\quad y_{ij}\ge 0,\; \forall (i,j) \in A^\text {prod}. \end{aligned}$$

Here, we assume that all the costs are *separable* and *convex*, but not necessarily differentiable at each point, i.e., we account for non-smooth cost functions often arising due to loading and raw material switching effects, multiple production machines with different costs etc. Such functional forms imply high flexibility in terms of cost structures accounted for by our model. Note that we do not impose lower bounds on the inventory arcs allowing for backlogging effects. This is in line with the assumption of convexity of the cost function associated with inventory: in particular, the function can be monotonically increasing in the positive range but monotonically decreasing in the negative range which signifies higher costs as inventory becomes negative and drops further, i.e., in case of high backlog amounts which can only occur if production capacities are too low in certain periods.

### Regret function

Importantly, problem (1) does not take production vulnerability into account, as reflected in the model presented in Snyder et al. (2006). Obtaining an optimal flow in this problem is equivalent to obtaining an optimal production plan in the problem without disruptions. In order to model production disruptions and to extend the problem to a stochastic one, we further introduce the *regret function*.

**Fig. 2:**
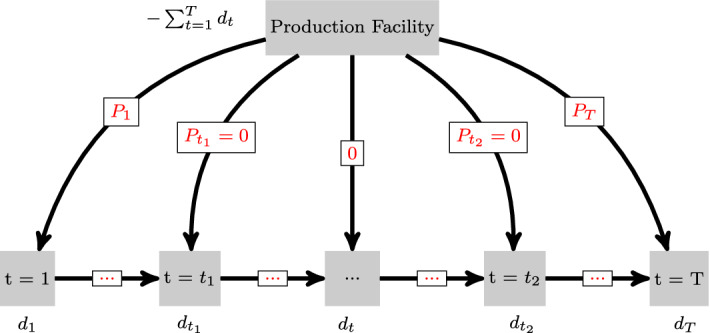
Multi-period production scheme disrupted from period $t_1$ to $t_2$

Denote the set of all disruption scenarios by $\varOmega$ and assume that $\omega _0 \in \varOmega$ is the scenario with no disruption. For every other scenario $\omega \in \varOmega \setminus \{\omega _0\}$ assume that some production arcs are disrupted according to a discrete-time Markov process, where each scenario has a probability $p_{\omega }$ with $\sum _{\omega \in \varOmega } p_{\omega } = 1$. Specifically, disruption and recovery probabilities $\alpha$ and $\beta$ are given such that in any period there is a chance of a disruption beginning and consequently concluding in any following period. We only consider scenarios where once the disruption recovers the facility will not become disrupted again until the end of the planning horizon. This is due to the fact that the likelihood of multiple non-consecutive disruptions is small (see Sect. 5). We assume that a production disruption during some period *t* changes the convex cost function in the corresponding arc to some other arbitrary convex cost function. This formulation incorporates many cases of interest, such as drops in production capacity (since the production capacity constraints can be pushed into the objective function while preserving convexity) or increases in production costs. Figure 2 demonstrates a production which is fully disrupted from period $t_1$ to $t_2$ (i.e., the production capacity drop to 0). Defining the loss incurred by a decision-maker as some production disruption occurs, we incorporate it into our model.

#### Definition 1

Let $A^{\text {prod}}(\omega )$ be the subset of disrupted production arcs in scenario $\omega$ and let $A^{\text {fix}}(\omega )$ contain all the arcs for which the flow is not modified in this scenario (i.e., the arcs prior to the disruption). The *regret function* is the distance between optimal values of an *anticipative* and a *reactive* cost minimization problem:

2 $$\begin{aligned} R(y,\omega ) := v^{\text {react}}(y,\omega ) - v^{\text {anticipate}}(\omega )\ge 0, \end{aligned}$$

where

3 $$\begin{aligned} v^{\text {anticipate}}(\omega )= & {} \mathop {\min }\limits _{x}\quad {\sum _{(i,j) \in A^{}(\omega )} {C}_{ij}(x_{ij}) }\nonumber \\&\text {s.t.}\qquad {\sum _{(j,i) \in A} x_{ji}^{} - \sum _{(i,j) \in A} x_{ij}^{}=d_i,\; \forall \; i \in V}\nonumber \\&\qquad \quad {x_{ij}\ge 0,\; \forall \; (i,j) \in A^\text {prod},\quad x_{ij}= 0,\; \forall \; (i,j) \in A^\text {prod}(\omega )}.\nonumber \\&\qquad \quad {x_{ij}\le \kappa _{ij},\; \forall \; (i,j) \in A,} \end{aligned}$$

and

4 $$\begin{aligned} \begin{array}{ll} v^{\text {react}}(y,\omega ) &{}{=} \mathop {\min }\limits _{x}\quad {\sum \limits _{(i,j) \in A^{}(\omega )} {C}_{ij}(x_{ij}) }\\ &{}\quad \text {s.t.}\qquad {\sum \limits _{(j,i) \in A} x_{ji}^{} - \sum \limits _{(i,j) \in A} x_{ij}^{}=d_i,\;\forall \; i \in V}\\ &{}\qquad \quad {x_{ij}\ge 0,\; \forall \; (i,j) \in A^\text {prod},\quad x_{ij}= 0,\; \forall \; (i,j) \in A^\text {prod}(\omega )}\\ &{}\qquad \quad {x_{ij}= y_{ij},\; \forall \; (i,j) \in A^\text {fix}(\omega )}\\ &{}\qquad \quad {x_{ij}\le \kappa _{ij},\; \forall \; (i,j) \in A,} \end{array} \end{aligned}$$

where the disrupted flow in the network is denoted by $x = (x_{ij})_{(i,j) \in A}$. Importantly, the flow can be negative for inventory arcs if demands are not met, i.e., the flow $x$ incorporates possible backlogs satisfied once enough goods are produced.

Note that the *anticipative plan* in Definition 1 represents the best possible strategy that could have been executed if the realized disruption scenario $\omega$ and its ending period $t_2$ would be known at the beginning of the planning horizon. In contrast, the *reactive plan* realistically assumes that the decision-maker learns about the disruption scenario $\omega$ at the moment when the disruption starts. At this point in time, the decision-maker is allowed to re-plan without modifying past production and inventory decisions. Thus, the reactive strategy consists of a plan *y*, which is decided at the beginning of the planning horizon and is executed until the disruption starts, and an updated plan $x$, developed in period $t_1$. In the reactive setup, the decision-maker is assumed to have an estimate for the ending period $t_2$ of the disruption $\omega$ once the disruption starts at time $t_1$.

**Fig. 3:**
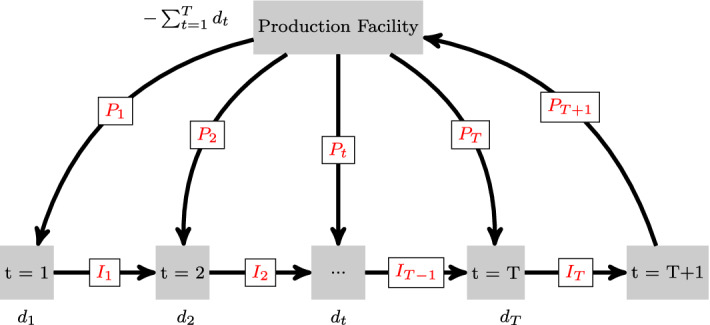
Extension with dummy node

Further note that disruptions may cause optimization problems (3) and (4) to become infeasible if there is no solution in which the backlog can be resolved by the final period. We avoid this issue by introducing a dummy node $T+1$, a dummy production arc $(0,T+1)$ and a dummy inventory arc $(T,T+1)$ into the network (see Fig. 3). The cost function corresponding to the dummy production arc is identical to 0, while the cost in the dummy inventory arc is a sufficiently large penalty that is paid for any backlog remaining at the end of the planning horizon. Finally, capacity constraints for these dummy arcs are chosen to be sufficiently large, such that feasibility is guaranteed for any possible disruption scenario $\omega$.

In our setup, the decision-maker aims for a lower value of $R(y,\omega )$, implying that the production plan should account for possible deviations in the manufacturer’s strategy from the idealistic but not realizable anticipative strategy to the worst-case reactive plan. The regret is measured in relation to the benchmark anticipative plan as it evaluates how fine the production schedule could be if the disruption would be known at the start of the planning horizon. Imposing an upper bound on the value of the regret provides several advantages compared to the straightforward approach in which the decision-maker simply minimizes the expected cost under all possible disruption scenarios. Firstly, it gives the opportunity to account for low probability disruptions which are typically poorly captured by expectation. Moreover, our approach allows to obtain solutions in which the amount of mitigation is risk-adjusted. This is due to the fact that extremely unlikely scenarios which require large investments in mitigation and present high regrets can still be avoided in the chance constraint.

Numerically, imposing additional constraints on the values of $R(y,\omega )$ for some scenarios $\omega \in \varOmega$ significantly complicates optimization problem (1) and makes it bilevel. This is because one needs to solve a sequence of optimization problems (3) and (4) in order to evaluate the regret $R(y,\omega )$ at any value pair $(y,\omega )$. On the one hand, one could approach the solution by imposing upper bounds on the bilevel optimization problem, as in Timonina-Farkas et al. (2020). On the other hand, the problem can be solved via decomposition methods with the aim of finding the *optimal resilient plan* $y^*$ that minimizes the total cost and also satisfies these additional constraints. We follow this path in Sect. 4.

### Chance-constraint formulation

To account for production disruptions in problem (1), we introduce a chance constraint on the regret function $R(y,\omega )$ into the model. Since our goal is to obtain a minimal cost strategy $y^*$ so that production breakdowns are sufficiently mitigated, we incorporate a chance constraint in the form ${\mathbb {P}}(R(y,\omega ) \le \gamma )\ge 1 - \theta$, requiring that the regret does not exceed some threshold $\gamma$ with probability $1-\theta$, where the *risk budget* $\theta$ corresponds to the sum of probabilities of scenarios with regret larger than the threshold. Thus, we obtain the following optimization problem:

5 $$\begin{aligned} \begin{array}{ll} {\hat{v}}&{}= \mathop {\min }\limits _{y}\quad { \sum \limits _{(i,j) \in A} {\widetilde{C}}_{ij}(y_{ij}) }\\ &{}\qquad \text {s.t.}\quad {\sum \limits _{(j,i) \in A} y_{ji}^{} - \sum \limits _{(i,j) \in A} y_{ij}^{}=d_i,\forall \; i \in V}\\ &{}\qquad \qquad {{\mathbb {P}}(R(y,\omega ) \le \gamma )\ge 1 - \theta .} \end{array} \end{aligned}$$

Here, we replace cost functions $C_{ij}(y_{ij})$ from the problem (1) by transformed cost functions ${\widetilde{C}}_{ij}(y_{ij})$ into which all upper and lower bound constraints are incorporated. Specifically, let $\delta _{+}(z)$ be an indicator function taking value 0 if $z\ge 0$ and $+\infty$ otherwise. Then the transformation ${\widetilde{C}}_{ij}(y_{ij}) = C_{ij}(y_{ij}) + \delta _{+}(\kappa _{ij} - y_{ij})$ retains upper bounds on $y_{ij}$ while preserving convexity of the objective function. We deal with lower bound constraints (including non-negativity constraints) using a similar indicator function $\delta _{-}(\cdot )$ which takes value 0 if $z\le 0$ and $+\infty$ otherwise, and preserves the convexity of the objective function as well.

The optimal solution $y^*$ of problem (5) is the *resilient plan*. The difference between the optimal values *v* of (1) and ${\hat{v}}$ of (5) is the *cost to mitigate* the disruption scenario set $\varOmega$. Introducing a sufficiently large constant *M*, we reformulate problem (5) into its deterministic equivalent:

6a $$\begin{aligned}&\mathop {\min }\limits _{y}\quad { \sum _{(i,j) \in A} {\widetilde{C}}_{ij}(y_{ij}) } \end{aligned}$$

6b $$\begin{aligned}&\text {s.t.}\quad {\sum _{(j,i) \in A} y_{ji}^{} - \sum _{(i,j) \in A} y_{ij}^{}=d_i,\; \forall \; i \in V} \end{aligned}$$

6c $$\begin{aligned}&\quad \qquad {R(y,\omega )\le Mz_{\omega } + \gamma ,\; \forall \; \omega \in \varOmega } \end{aligned}$$

6d $$\begin{aligned}&\quad \qquad {\sum _{\omega \in \varOmega } p_{\omega }z_{\omega }\le \theta } \end{aligned}$$

6e $$\begin{aligned}&\quad \qquad {z_{\omega }\in \{0,1\},\; \forall \; \omega \in \varOmega ,} \end{aligned}$$

where *M* is a sufficiently large constant and $z_{\omega }$ are binary variables determining whether the regret of scenario $\omega$ exceeds the threshold $\gamma$. This problem is a mixed-integer non-linear problem (MINLP) and is solvable via existing tools such as BARON or BONMIN (Bussieck and Vigerske 2010) for small-dimensional cases. Furthermore, if the cost can be approximated by a piece-wise linear function, the problem can be reformulated as a mixed-integer linear problem (MILP) solvable using standard software such as Gurobi or CPLEX. However, it is well known that such reformulations involving a sufficiently large constant *M* can lead to computational difficulties. Selecting *M* as tightly as possible can mitigate these issues and result in an improved performance. In particular, upper bounds on the regrets of the most disadvantageous scenarios can be easily computed ahead of time and used to determine values for *M*. Nevertheless, as the number of decision variables grows, the cardinality of the scenario set becomes prohibitively large, making the problem complex to address beyond a planning horizon of $T=15$.

To solve the problem in higher dimensions, we develop an efficient solution algorithm. We use a generalized Benders procedure to decompose the complexity and solve a sequence of small-dimensional problems whose solutions converge to the optimum of the initial problem. For the convergence, we gradually add customized feasibility cuts to the sequence. The cuts incorporate information about the disruption scenarios without the introduction of additional variables and enable us to avoid the “curse of dimensionality” by solving many smaller problems rather than a single complex one. Although this is a classic idea in optimization, we adapt it to our problem by introducing customized feasibility cuts and avoiding optimality cuts. In the next section, we describe the procedure in detail.

## Solution method

A feasible point (*y*, *z*) satisfying constraints (6b), (6d) and (6e) is optimal in the problem (6a) if *y* minimizes the objective function and if $R(y,\omega ) \le Mz_{\omega } + \gamma$ holds for every scenario $\omega$. Note that one only needs to check scenarios $\omega$ such that $z_{\omega } = 0$, since scenarios $\omega$ with $z_{\omega } = 1$ obviously satisfy the constraint because $M$ is large. Next, the value of $v^{\text {anticipate}}(\omega )$ depends on scenario $\omega$ but not on the solution $y$ and, thus, it can be computed prior to solving the problem (6a). The subproblems (3) and (4) are independent of each other. In order to evaluate the regret function, we need to compute the objective value of the problem (4) for each scenario $\omega$ such that $z_{\omega } = 0$ and to check whether it is smaller than $v^{\text {anticipate}}(\omega ) + \gamma$. Inspired by the approach outlined in Zheng et al. (2015), we apply a generalized Benders decomposition first introduced in Geoffrion (1972). We customize the procedure by introducing efficient feasibility cuts to the following *relaxed master problem:*

7 $$\begin{aligned} \begin{array}{l} \mathop {\min }\limits _{y}\qquad {\sum \limits _{(i,j) \in A} {\widetilde{C}}_{ij}(y_{ij}) } \\ \text {s.t.}\qquad \quad {\sum \limits _{(j,i) \in A} y_{ji}^{} - \sum \limits _{(i,j) \in A} y_{ij}^{}=b_i,\forall \; i \in V}. \end{array} \end{aligned}$$

At each iteration *k*, the Benders procedure obtains a solution $(y^k,z^k)$ of the problem (7) with additional cuts and checks if the feasibility of $R(y^k,\omega ) \le \gamma$ holds for all $\omega$ such that $z_{\omega }^k = 0$. For each scenario violating the constraint, we add a feasibility cut to the optimization by solving the following *Benders feasibility problem*:

8 $$\begin{aligned} \begin{array}{ll} \varDelta _\text {Feas}^k(y^k, \omega )&{}=\mathop {\min }\limits _{\varDelta _{\omega }}\quad { | \varDelta _{\omega } | }\\ &{}\quad \text {s.t.}\quad {R(y^k,\omega ) - \gamma }{\le \varDelta _{\omega },} \end{array} \end{aligned}$$

where the optimal value $\varDelta _\text {Feas}^k(y^k, \omega )$ is the amount by which the constraint is violated. For all $\omega \;\text { such that}\; \varDelta _\text {Feas}^k(y^k, \omega ) > 0$, problem (8) is equivalent to:

9a $$\begin{aligned} \varDelta _\text {Feas}^k(y^k, \omega )= & {} \mathop {\min }\limits _{x}\quad { \sum \limits _{(i,j) \in A^{}(\omega )} {\widetilde{C}}_{ij}^{\text {react}}(x_{ij}) - v^{\text {anticipate}}(\omega ) - \gamma } \end{aligned}$$

9b $$\begin{aligned}&\text {s.t.}\quad {\sum _{(j,i) \in A} x_{ji}^{} - \sum _{(i,j) \in A} x_{ij}^{}=d_i \; \forall \; i \in V} \end{aligned}$$

9c $$\begin{aligned}&\qquad \quad {x_{ij}= 0 ,\; \forall \; (i,j) \in A^{\text {prod}}(\omega )} \end{aligned}$$

9d $$\begin{aligned}&\qquad \quad {x_{ij}= y_{ij}^k ,\; \forall \; (i,j)\in A^{\text {fix}}(\omega ).} \end{aligned}$$

Here, $\varDelta _\text {Feas}^k(y^k, \omega ) = v^{\text {react}}(y^k, \omega )-v^{\text {anticipate}}(\omega ) - \gamma$ and, thus, ${\widetilde{C}}_{ij}^{\text {react}}(x_{ij})$ is the cost function of optimization problem (4) incorporating its capacity and non-negativity constraints. Note that the objective function remains convex as the value $v^{\text {anticipate}}(\omega )$ is constant for each $\omega$.

The optimization problem (9a) is a convex minimum-cost network flow problem. Denoting by $\pi _i$ the dual variables corresponding to the flow equilibrium constraints (9b), we write the *complementary slackness conditions* (see Bertsekas 1998) as:

10 $$\begin{aligned} \partial _{-}{\widetilde{C}}_{ij}^{\text {react}}(x_{ij}) \le \pi _i - \pi _j \le \partial _{+}{\widetilde{C}}_{ij}^{\text {react}}(x_{ij}),\quad \forall (i, j)\in A, \end{aligned}$$

where $\partial _{-}$ and $\partial _{+}$ denote left and right derivatives respectively.

Further, let us denote by $x^*$ the primal optimal solution of the problem. Due to the well-known complementary slackness theorem (see Bertsekas 1998), $\pi _i$ and $\pi _j$ satisfying inequalities (10) for $x=x^*$ and every arc (*i*, *j*) are dual optimal. Moreover, as long as the problem has at least one primal feasible solution (which our problem does by construction), the optimal dual point $\pi = (\pi _i, \pi _j)$ satisfying the conditions exists (see Bertsekas 1998).

Next, we compute the optimal dual variables $\rho _{ij}^+$ and $\rho _{ij}^-$ for constraints $x_{ij}\ge y_{ij}^k$ and $x_{ij}\le y_{ij}^k$ corresponding to condition (9d) of the problem.

### Theorem 1

The values

$$\begin{aligned}\rho _{ij}^{+} = \left\{ \begin{array}{cc} max (0, \partial _{-}{\widetilde{C}}_{ij}^{react }(x_{ij}) - \pi _i + \pi _j ), &{} if (i,j) \in A\setminus A^{fix }(\omega )\\ max (0, - \pi _i + \pi _j ), &{} if (i,j) \in A^{fix }(\omega ) \end{array}\right. \\\rho _{ij}^{-} = \left\{ \begin{array}{cc} min (0, \partial _{+}{\widetilde{C}}_{ij}^{react }(x_{ij}) - \pi _i + \pi _j ), &{} if (i,j) \in A\setminus A^{fix }(\omega )\\ min (0, - \pi _i + \pi _j ), &{} if (i,j) \in A^{fix }(\omega ) \end{array}\right. \end{aligned}$$

are optimal dual multipliers for constraints $x_{ij}\ge y_{ij}^k$ and $x_{ij}\le y_{ij}^k$, which are jointly equivalent to the condition (9d).

### Proof

Consider the following problem, where we state all the constraints:

11a $$\begin{aligned}&\mathop {\min }\limits _{x}\quad { \sum _{(i,j) \in A(\omega )} C_{ij}^{\text {react}}(x_{ij}) - v^{\text {anticipate}}(\omega ) - \gamma } \end{aligned}$$

11b $$\begin{aligned}&\text {s.t.}\quad {\sum _{(j,i) \in A} x_{ji}^{} - \sum _{(i,j) \in A} x_{ij}^{}=d_i \; \forall \; i \in V} \end{aligned}$$

11c $$\begin{aligned}&\quad \qquad {x_{ij}\le \kappa _{ij},\; \forall (i,j) \in A} \end{aligned}$$

11d $$\begin{aligned}&\quad \qquad {x_{ij}\ge 0,\; \forall (i,j) \in A^\text {prod}} \end{aligned}$$

11e $$\begin{aligned}&\quad \qquad {x_{ij}= 0 ,\; \forall (i,j) \in A^{\text {prod}}(\omega )} \end{aligned}$$

11f $$\begin{aligned}&\quad \qquad {x_{ij}\le y_{ij}^k,\; \forall (i,j) \in A^{\text {fix}}(\omega )} \end{aligned}$$

11g $$\begin{aligned}&\quad \qquad {x_{ij}\ge y_{ij}^k,\; \forall (i,j) \in A^{\text {fix}}(\omega )}. \end{aligned}$$

Denote by ${\tilde{F}}$ the feasible region defined by all constraints except the network flow equilibrium constraint and the fixing constraints, i.e., ${\tilde{F}} = \{ x \mid \;$(11c), (11d), (11e)$\}$. Dualizing the problem on constraints (11b), (11f) and (11g), we obtain the Lagrangian:

$$\begin{aligned}&L(x,\pi ,\rho _{ij}^{+},\rho _{ij}^{-}) = \sum _{(i,j) \in A(\omega )} C_{ij}^{\text {react}}(x_{ij}) - v^{\text {anticipate}}(\omega ) - \gamma \\&\quad + \sum _{i \in N} \pi _i \bigg (\sum _{(j,i) \in A} x_{ji}^{} - \sum _{(i,j) \in A} x_{ij}^{} - d_i\bigg ) + \sum _{(i,j) \in A} \rho _{ij}^{+}(x_{ij} - y_{ij}^k) \\&\quad + \sum _{(i,j) \in A} \rho _{ij}^{-}(y_{ij}^k - x_{ij})\end{aligned}$$

and the resulting dual problem is:

12 $$\begin{aligned}&\mathop {\max }\limits _{\pi ,\rho _{ij}^{+},\rho _{ij}^{-}} \quad {\inf \limits _{x \in {\tilde{F}}} \quad L(x,\pi ,\rho _{ij}^{+},\rho _{ij}^{-})}\nonumber \\&\text {s.t.}\quad {\rho _{ij}^{-} \le 0, \;\; \rho _{ij}^{+} \ge 0.}{} \end{aligned}$$

Dual variables $\pi$ satisfying (10) are optimal for (12). Thus, taking the subgradient on $x_{ij}$ of the Lagrangian leads to the following optimality conditions:

13 $$\begin{aligned} 0 \in \partial _{ij} C_{ij}^{\text {react}}(x_{ij}) - \pi _i + \pi _j + \rho _{ij}^{+},\quad \forall (i,j) \in A\setminus A^{\text {fix}} \end{aligned}$$

and

14 $$\begin{aligned} 0 \in - \pi _i + \pi _j + \rho _{ij}^{+},\quad \forall (i,j) \in A^{\text {fix}}(\omega ). \end{aligned}$$

The variable $\rho _{ij}^{+}$ acts as a penalty in the dual problem objective: Larger values of $\rho _{ij}^{+}$ lead to a lower objective value. Thus, maximization implies a value of $\rho _{ij}^{+}$ that is as small as possible. Due to convexity and non-negativity constraints, this value is either at the boundary of the feasible region (i.e., is equal to 0) or is such that (13) or (14) is satisfied. The subgradient of the cost function increases monotonically because of the function’s convexity. At any point $x_{ij}$, the left derivative takes the smallest possible value. Thus, we may set:

$$\begin{aligned}\rho _{ij}^{+} = \left\{ \begin{array}{cc} \text {max}(0, \partial _{-}C_{ij}^{\text {react}}(x_{ij}) - \pi _i + \pi _j ), &{} \text {if }(i,j) \in A\setminus A^{\text {fix}}(\omega )\\ \text {max}(0, - \pi _i + \pi _j ), &{} \text {if }(i,j) \in A^{\text {fix}}(\omega ). \end{array}\right. \end{aligned}$$

Similarly, we obtain the result for $\rho _{ij}^{-}$. $\square$

The multipliers resulting from inequalities (10) and Theorem 1 are used to construct Benders feasibility cuts (Fischetti et al. 2016; Geoffrion 1972) in the following way:

15 $$\begin{aligned} Mz_{\omega } \ge \varDelta _\text {Feas}^k(y^k, \omega ) + \sum _{(i,j) \in A^{\text {fix}}(\omega )} (\rho _{ij}^{+} + \rho _{ij}^{-})(y_{ij} - y_{ij}^k) \end{aligned}$$

The term $Mz_{\omega }$ controls whether the cut corresponding to scenario $\omega$ is active at iteration $k$ of the Benders procedure. Note that the sequence of optimal values is non-decreasing, as we add more constraints at each iteration. The derivation of dual solutions for Benders feasibility cuts remains true if we consider a disruption scenario $\omega$ to represent a change in a convex cost function $C_{ij}(\cdot )$ in affected arcs $(i,j) \in A^{\text {prod}}(\omega )$ in subproblems (3) and (4). This allows us to model the *agility capacity* (see Tang 2006), where partial production remains during a disruption, possibly at a higher cost. The resulting procedure is presented in Algorithm 1.
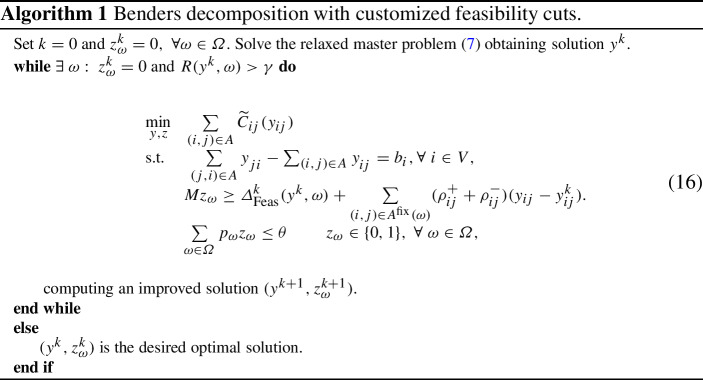


Note that the solution $(y^0,z_{\omega }^0)$ implies a lower bound on the optimal value of the problem (6a): $y^0$ is the optimal solution of the non-perturbed optimization problem (1) and $z_{\omega }^0$ is a feasible point of constraints (6d) and (6e). In Algorithm 1, we compute the regret $R(y^k, \omega ) = v^{\text {react}}(y^k, \omega ) - v^{\text {anticipate}}(\omega )$ for each scenario $\omega$ such that $z^k_{\omega }= 0$ via solving lower-level problems (3) and (4) with $y=y^k$.

## Scenario generation

We employ two scenario generation methods in order to construct the set of scenarios $\varOmega$ in Algorithm 1. The first method assumes equally probable disruptions of the same length throughout the planning horizon $[1,\ldots ,T]$, i.e., using $\phi \in [0,\; 1]$ to denote the probability of a disrupted period, we obtain the probability of no disruption as $(1-\phi )^T$. Normalizing the values and avoiding non-consecutive disruptions, we obtain the distribution function. See Torabi et al. (2016), Hatefi and Jolai (2014) and Hatefi et al. (2015) for similar approaches.

The other method utilizes probabilities of a discrete-time infinite-state Markov process (see Schmitt et al. 2010, 2015) for modeling production disruptions. In this approach, which is often used to model disruptions in newly built production sites, we use states with $s\ge 1$ to denote the number of consecutively disrupted time periods, $\alpha$ to denote the transition probability from a state with no disruption to a disrupted state for each time period (i.e., the *disruption probability*) and $\beta$ to denote the transition probability from a state with $s\ge 1$ disrupted periods to a state with no disruption (i.e., the *recovery probability*). We further assume that these probabilities are independent. The steady state probabilities are defined by Schmitt et al. (2010) as

$$\begin{aligned} \xi _0 = \frac{\beta }{\alpha + \beta }, \qquad \xi _s = \frac{\alpha \beta }{\alpha + \beta }(1-\beta )^{s-1},\; s\ge 1, \end{aligned}$$

where $\xi _0$ is the probability of no disruption and $\xi _s$ is the probability of $s\ge 1$ disrupted periods. Given $\alpha$ and $\beta$, the mean and the variance of the duration are

$$\begin{aligned} {\mathbb {E}}(s) = 0\cdot \xi _0+\sum _{s=1}^{\infty }s\xi _s = \frac{\alpha }{(\alpha +\beta )\beta },\qquad {\mathbb {V}}ar(s)={\mathbb {E}}^2(s)\bigg (1+2\frac{\beta }{\alpha }\bigg )-{\mathbb {E}}(s), \end{aligned}$$

where we use the sum $\big (\frac{\alpha }{(\alpha +\beta )\beta }\big )^2\xi _0+\sum _{s=1}^{\infty }\big (s-\frac{\alpha }{(\alpha +\beta )\beta }\big )^2\xi _s$ to derive the expression for the variance.

Besides the production with infinite possible states, we can also model finite-state disruptions happening during finite planning horizons using the probabilities $\alpha$ and $\beta$. In particular, under the assumption that only one disruption may take place during our finite planning horizon, the following Lemma holds:

### Lemma 1

The probability that the site is disrupted at some period $t \in \{1,\ldots ,T\}$ is equal to

$$\begin{aligned} p_{t}(\alpha ,\beta ) = \frac{\alpha }{\alpha + \beta }\left( \beta \frac{(1-\alpha )^t - (1-\beta )^{t}}{(1-\alpha )-(1-\beta )} + (1-\beta )^{t} \right) . \end{aligned}$$

### Proof

To derive $p_{t}(\alpha ,\beta )$, we sum up probabilities of two separate cases: one under which a disruption starts at the beginning of the planning horizon and another under which it begins later. From the first case one obtains the disruption probability $(1 - \xi _0)(1-\beta )^t$. Conversely, in the second case we let $u \le t$ be the period in which a disruption starts. Summing over all possible periods we obtain the disruption probability

$$\begin{aligned}\xi _0 \sum _{u=1}^{t}(1-\alpha )^{u-1}\alpha (1-\beta )^{t-u} =\xi _0 \alpha \left( \frac{(1-\alpha )^t - (1-\beta )^{t}}{(1-\alpha )-(1-\beta )}\right) , \end{aligned}$$

where the right-hand side is obtained using the identity $\sum _{j=0}^{n}a^{j}b^{n-j} = \frac{a^{n+1}- b^{n+1}}{a-b}$. Note that this identity can be proven by induction for $0 \le a,b \le 1$ and $n \in {\mathbb {N}}_0$. $\square$

**Fig. 4:**
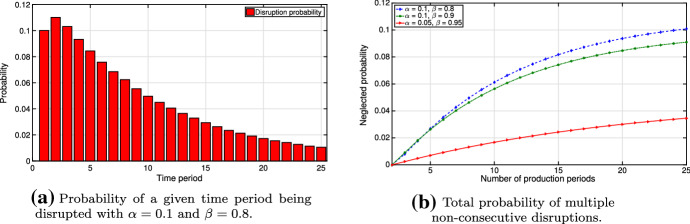
Analysis of disruption probabilities

Now, given a finite time horizon with $T$ periods, we simulate production disruptions and demonstrate estimated probabilities in Fig. 4. Figure 4a shows the probability of a particular production period to be disrupted for $T=25$, while Fig. 4b demonstrates the neglected probability, based on the fact that non-consecutive disruptions are being omitted.

In Fig. 4, we observe that early production periods are more likely to be disrupted. Although disruptions starting in later production periods tend to be less probable, they lead to higher losses because of our definition of the regret function (2) and too little time left for recovery. Note that the values of $\alpha$ and $\beta$, which are the inputs in our model, define the expected disruption duration and the expected starting period. Further, Fig. 4b shows that the total neglected probability of more than one non-consecutive disruption decreases if $\alpha$ decreases and $\beta$ increases. Setting appropriate values for $\alpha$ and $\beta$ in our model, we focus only on consecutive production disruptions within our planning horizon, limiting the neglected probability to less than 1%, which is below the red line in Fig. 4b. In practice, appropriate values of $\alpha$ and $\beta$ can be determined via statistical approaches through the use of historical data collected based on past events. This is also possible in case of rare events as described by Amrein (2011) for Markov processes. Additionally, panel data may be used, i.e., expert practitioners may be polled to determine adequate values.

## Functional properties

Our goal in this section is to gain an understanding on how the optimal value function ${\hat{v}}(\gamma ,\theta )$ of (5) and the corresponding optimized regret $R(y, \omega )$ behave depending on our choice of parameters $\gamma$ and $\theta$, as well as on the scenario set $\varOmega$.

### Lemma 2

The following monotonicity conditions hold for the optimal value function ${\hat{v}}(\cdot )$ of the optimization problem (5) in risk budget parameters:

(i) $${\hat{v}}(\gamma ,\theta ,\varOmega ) \ge {\hat{v}}(\gamma ',\theta ,\varOmega ), \;\text { if }\; \gamma ' \ge \gamma $$
(ii) $${\hat{v}}(\gamma ,\theta ,\varOmega ) \ge {\hat{v}}(\gamma ,\theta ',\varOmega ), \;\text { if }\; \theta ' \ge \theta $$.

### Proof

**Fig. 5:**
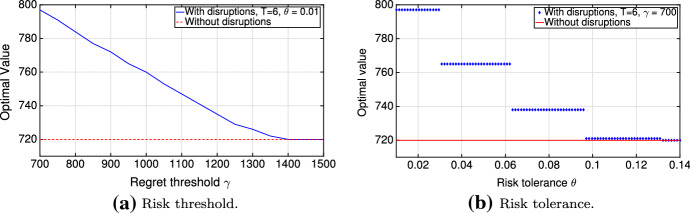
Sensitivity of the optimal value to parameters

Smaller or equal feasible regions for lower values of $\gamma$ and $\theta$ suggest that statements (i) and (ii) for the minimization problem (5) hold (Fig. 5a, b). Thus, Lemma 2 holds due to the fact that any optimal solution in a more restricted problem remains feasible in a less restricted one. $\square$

The monotonicity conditions of Lemma 2 are very important as they describe the behavior of the optimal value function in comparison to the risk threshold and risk tolerance (i.e., risk budget parameters): in particular, the optimal cost decreases if risk budget parameters increase. Also, following from the discreteness of the disruption scenario process, the optimal value function ${\hat{v}}(\gamma ,\theta ,\varOmega )$ of the optimization problem (5) is a step function in the risk tolerance parameter $\theta$ (Fig. 5b).

**Fig. 6:**
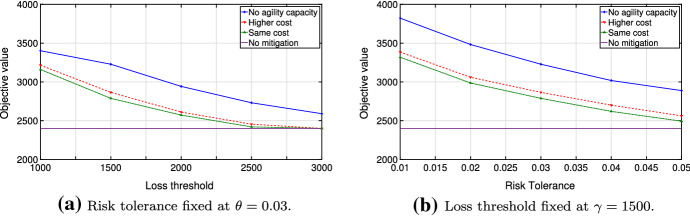
Objective values for disruption probability $\alpha = 0.05$ and recovery probability $\beta = 0.5$

**Fig. 7:**
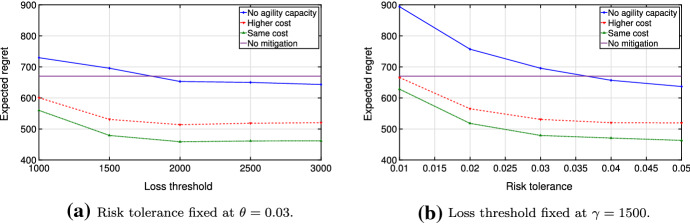
Expected regrets for disruption probability $\alpha = 0.05$ and recovery probability $\beta = 0.5$

Analyzing the function ${\hat{v}}(\gamma ,\theta ,\varOmega )$ in more detail, we compare disrupted and undisrupted cases and perform extensive numerical tests for piece-wise linear cost functions. For this, we focus on the following three cases:

1. No production capacity is available during disrupted periods;
2. Agility capacity is available at a regular cost during disrupted periods;
3. Agility capacity is available at a higher cost during disrupted periods (i.e., a five-fold cost increase in Figs. 6 and 7 ).

Our model formulation supports examination of all three cases, as well as any other situation in which a disruption may be modeled as a change of a convex cost function to another convex cost function. In our further analysis, we consider an example in which the available agility capacity corresponds to one-sixth of regular production capacity and the scenario set is constructed in line with the Markov process outlined in Sect. 5 with $\alpha = 0.05$ and $\beta = 0.5$.

Figure 6 demonstrates the optimal value of the problem (5) with $T=20$, $\theta = 0.03$ and $\gamma =1500$. Note that the cost function $v \le {\hat{v}}(\gamma ,\theta ,\varOmega )\; \forall \gamma , \theta , \varOmega$, where *v* is the optimal value of the undisrupted problem (1). This relationship follows from the fact that the total cost decreases as we relax the problem, since less inventory is needed early in the planning horizon. Also, the agility capacity lessens the amount of the mitigation inventory needed and further decreases costs. Thus, the optimal cost tends to *v* if risk parameters in the chance constraint of problem (5) increase (Fig. 6).

Nevertheless, the optimal value alone cannot provide complete information about the quality of a solution. Therefore, we study the resilient plan, analyzing the behavior of the optimized regret and comparing it to the case with no mitigation. Specifically, we consider the *expected regret* ${\mathbb {E}}(R(y,\varOmega )) = \sum _{\omega \in \varOmega } p_{\omega }R(y,\omega )$ and its corresponding variance. Since disruptions are presumed to be rare and the probability of no disruptions $p_{\omega _0}$ is typically very high, one would expect a significant fraction of the value of the expected regret to result from $R(y,\omega _0)$ (regret of no mitigation plan). However, the disrupted scenarios contribute to the value of ${\mathbb {E}}(R(y,\varOmega ))$ to such an extent that one would be better off if one had invested in mitigation. In Fig. 7, we compare the expected regret under mitigation and no mitigation plans. We compute the expectation by considering scenario-specific regrets and the corresponding probabilities. Compared to the case with no mitigation, the expected regret tends to decrease in the presence of agility capacity under the optimized mitigation plan. Moreover, the under-performance of the mitigation plan becomes less likely when the disruption probability gets higher.

**Fig. 8:**
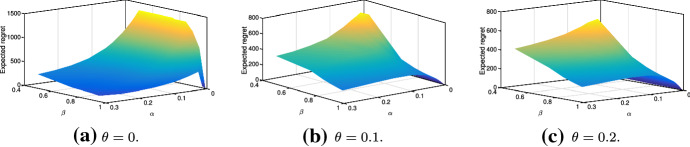
Expected regret for risk tolerances $\theta \in \{0,0.1,0.2\}$

Following this example, we account for risk tolerance levels $\theta \in \{0,0.1,0.2\}$ in Fig. 8. Here, the highest expected regrets are observed for low-probability long-lasting disruptions. For $\theta = 0$ (i.e., for the case with no risk tolerance), Fig. 8a shows a sharp increase in expected regrets of low-probability disruptions. This is due to the fact that every scenario must be mitigated irrespective of its likelihood and cost if $\theta = 0$. Thus, a large amount of resources is invested into mitigation and, therefore, high regrets can be observed in low-probability cases. Conversely, once $\theta > 0$ as in Fig. 8b, c, some low-probability scenarios may become tolerated in line with our model. By this, the total amount invested in the mitigation drops. Although we still observe high expected regrets for low-probability persistent disruptions, these values are lower than in the case with $\theta = 0$ (Fig. 8).

**Fig. 9:**
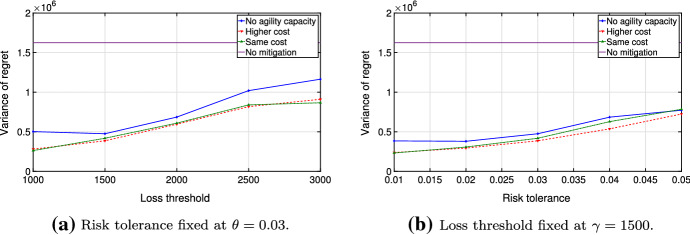
Variance of regrets for disruption probability $\alpha = 0.05$ and recovery probability $\beta = 0.5$

Once the expected regret is computed, the variance of the regret is evaluated and turns out to be lower in the mitigation plan $y$ than in the plan assuming no mitigation (Fig. 9). This is because any mitigation plan reduces regrets incurred by highly disrupted scenarios. Also, in addition to the variance, one may consider a mean-variance risk measure $Z(y) = {\mathbb {E}}(f(y)) - k{\mathbb {V}}\text {ar}(f(y))$ with a factor $k \ge 0$ used to control the risk-bearing ability of the decision-maker and a function *f* representing either the objective function or the regret (see Markovitz 1952). If the decrease in variance is large enough with respect to no mitigation plan $y_0$, it follows that $Z(y) < Z(y_0)$ even for small values of the parameter *k* with $k=0$ representing the risk-neutral case.

**Fig. 10:**
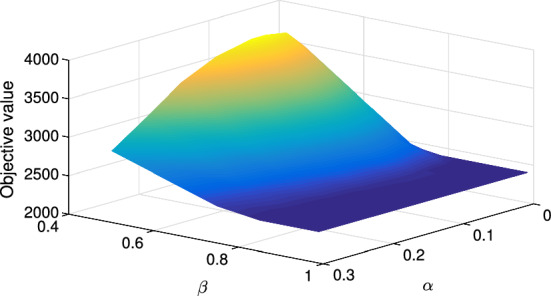
Objective values with threshold $\gamma = 1500$ and risk budget $\theta = 0.01$

Further, we examine the behavior of the optimal objective value of the problem (5) dependent on probabilities $\alpha$ and $\beta$, testing the same values as Schmitt et al. (2015). Figure 10 demonstrates the optimal value dependent on $\alpha$ and $\beta$ given the risk threshold $\gamma = 1500$ and risk budget $\theta = 0.01$.

If the recovery probability $\beta$ is high, all disruptions tend to be short and, therefore, not particularly costly. If their cost does not exceed the given threshold $\gamma = 1500$, no mitigation is required. Thus, the resilient plan coincides with the no mitigation plan and takes the same objective value of 2400. Similarly, if the disruption probability $\alpha$ is high, imminent disruptions tend to have lower regrets and do not require much mitigation. This is because (1) we assume only one disruption within the planning horizon, (2) the probability of a disruption starting in period *t* is $(1-\alpha )^{t-1}\alpha$ and, thus, higher $\alpha$ implies an earlier start of the disruption, on average, and (3) the regret function measures the unattained cost reduction of preparing for a disruption. As a consequence, the optimal objective value decreases as the disruption probability $\alpha$ increases (Fig. 10). Nevertheless, higher regrets and stronger mitigation plans can be expected if both the disruption and the recovery probabilities are not very high. We consider the influence of the start and the length of a disruption on the mitigation plan in detail in the next section.

## Managerial insights

**Fig. 11:**
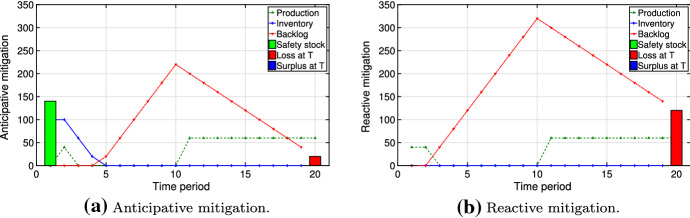
Anticipative vs. reactive mitigation for early/long disruptions

**Fig. 12:**
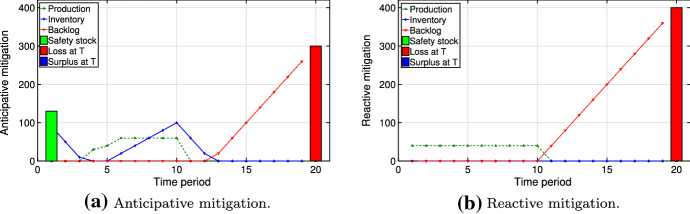
Anticipative vs. reactive mitigation for middle start/long disruptions

**Fig. 13:**
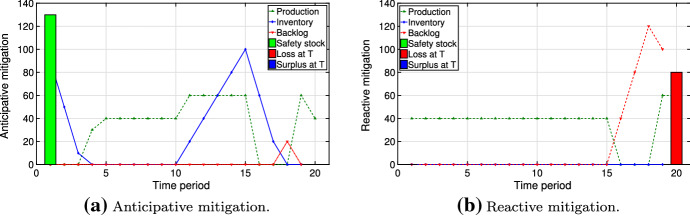
Anticipative vs. reactive mitigation for disruptions starting late in time

In this section, we present managerial insights related to the structure of the resilient plan under various conditions. We consider multiple types of disruption scenarios and their effect on the resilient plan *y* obtained by solving our optimization problem (5). We characterize scenario $\omega \in \varOmega$ by its starting period $t_{\omega }^{\text {start}}$ and its ending period $t_{\omega }^{\text {end}}$. The probabilities of scenarios $p_{\omega }\; \forall \omega \in \varOmega$ are a consequence of the Markov model presented in Sect. 5. The value of the regret is implicitly dependent on the probability $p_{\omega }$. Clearly, scenarios with higher regret govern the structure of the resilient plan: They either enter the risk budget (i.e., $z_{\omega }=0$) or are strongly mitigated. The scenario types are:

(i) **Early/long disruptions** (scenarios in which the disruption starts early and persists, $\alpha = 0.5$ and $\beta = 0.2$): Inventory amounts obtained via problems (3) and (4) are close to each other, as the time to prepare for early disruptions is short. Furthermore, the backlogging cost is high, as the disruptions are long. Thus, the regret tends to be high. Also, the cumulative probability of such scenarios is high due to the large number of them. As a result, mitigation for such scenarios creates an early peak in inventory level. Figure 11 shows this via comparing anticipative and reactive mitigation plans. The inventory helps to reduce the loss at time $T$ and the overall backlog. In the best case achieved via our simulation, the anticipative solution results in a 46% cost reduction computed by comparing optimal values for anticipative and reactive problems.
(ii) **Middle start/long disruptions** (scenarios with persisting disruptions starting near the middle of the horizon, $\alpha = 0.1$ and $\beta = 0.2$): Although such scenarios have the highest regrets, they are likely not to be mitigated. On the one hand, much time is available to prepare for the disruptions and, thus, the difference between anticipative and reactive plans is large. On the other hand, there is likely a penalty for any remaining backlog at the end of the planning horizon due to the persistence of the disruptions. However, the cumulative probability of middle start/long disruptions is low. Thus, they are likely to be selected as scenarios to be covered by the risk budget ($z_{\omega }=1$).
  Figure 12 compares anticipative and reactive mitigation plans for middle start but lasting disruptions, demonstrating high losses for both anticipative and reactive plans at time $T$. The anticipative mitigation plan in Fig. 12a results in a 31% cost reduction compared to the reactive plan in Fig. 12b.
(iii) **Late disruptions** (scenarios in which the disruption starts (and ends) late, $\alpha = 0.05$ and $\beta = 0.2$): Both factors from the previous case also apply here, making these scenarios rather costly. Furthermore, they typically have higher probability than middle start/long disruptions. Thus, they are less likely to enter the risk budget and the decision-maker mitigates them by creating a late peak in mitigation inventory (Fig. 13a). Due to the late start of such disruptions, Fig. 13 demonstrates a 100% decrease in losses at time $T$ in the anticipative plan: This is achieved by an increase in safety stock and inventory level before the disruption. The anticipative mitigation plan shown in Fig. 13a reduces the total cost by half in comparison to the reactive plan in Fig. 13b. This is computed by comparing the optimal values of the corresponding solutions.

As described above, the occurrence of scenarios (i)–(iii) in our model depends on the choice of the disruption probability $\alpha$ in the Markov process. Furthermore, changing the recovery probability $\beta$ one could influence the disruption length. Clearly, the optimal resilient plan $y$ depends both on the disruption scenarios and on the risk parameters $\theta$ and $\gamma$.

**Fig. 14:**
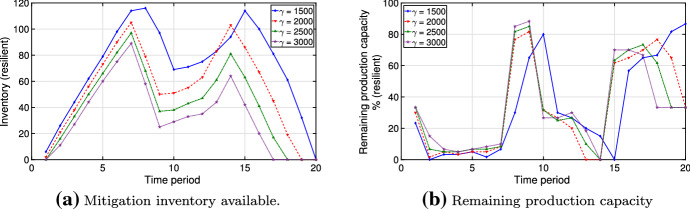
Example of risk clustering behavior for $\theta = 0.01$

For example, Figure 14a demonstrates the resilient plan given the risk tolerance parameter $\theta = 0.01$. The optimal solution requires higher levels of inventory in early and later production periods. Figure 14b displays the corresponding production capacity utilization. Clearly, high capabilities are required while mitigation inventory is being built up, allowing production to decline sharply prior to the drop in inventory. We refer to the behavior shown in Fig. 14 as to *risk clustering*, due to the fact that some types of scenarios are being tolerated with respect to other ones. In particular, the vast majority of scenarios of type (ii) are being selected into the risk budget and, therefore, tolerated in the example of Fig. 14. Thus, we observe higher inventories for early and late disruptions. Overall, particularly costly and rare disruptions are likely to be selected into the risk budget with lower or no mitigation being optimal for them. We observe this effect in the middle of the planning horizon in Fig. 14.

In practice, one may desire the variability of the optimal solution $y$ observed in Fig. 14 in cases with production shutdowns in a particular time of the year (e.g., summer). Vice versa, some manufacturers may want to avoid such a variability aiming for a stable production and inventory plan with a constant machine workload. Thus, note that the solution would be less volatile if the inventory were more costly. It would also be the case if the convex cost functions would have a higher curvature or if the capacities would be lower, which would reduce the distance between optimal values $v^{\text {react}}(y^*,\omega )$ and $v^{\text {anticipate}}(\omega )$ decreasing the regret values and the overall mitigation. Also, one could impose different regret thresholds depending on the starting period of a disruption.

We observe mitigation patterns in Figs. 8 and 14 for both scenario generation methods outlined in Sect. 5. Similar to the Markov model, regret in scenarios with a late disruption start remains high for the uniform case. However, if we only incorporate the costs generated by cumulative regrets after the disruption starts, risk clustering behavior is not observed in the case of linear cost functions and uniform probabilities. Instead, the resilient plan stabilizes at some inventory level, making this less of a concern for many practical applications. A full analysis of risk clustering behavior is a subject for future research.

**Fig. 15:**
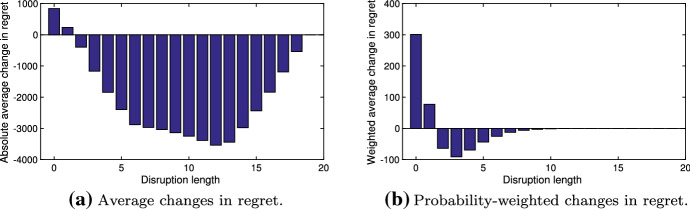
Change in regret between unmitigated and resilient plans

Importantly, the optimal solution $y$ produced by our methodology provides lower regrets even for scenarios in the risk budget ($\omega :\; z_{\omega }=0$): This is one of the key differences with other approaches based on the value-at-risk. The result is intuitive, since any mitigation is better than no mitigation in the case of a significant disruption. Conversely, mitigation increases the regret for short disruption scenarios, whose regret under no mitigation is typically far below the threshold $\gamma$ and, thus, its increase is optimal for the solution. Figure 15 demonstrates this behavior, outlining the dependence between (1) the average change in regret compared with no mitigation solution and (2) disruption lengths (in number of periods).

**Fig. 16:**
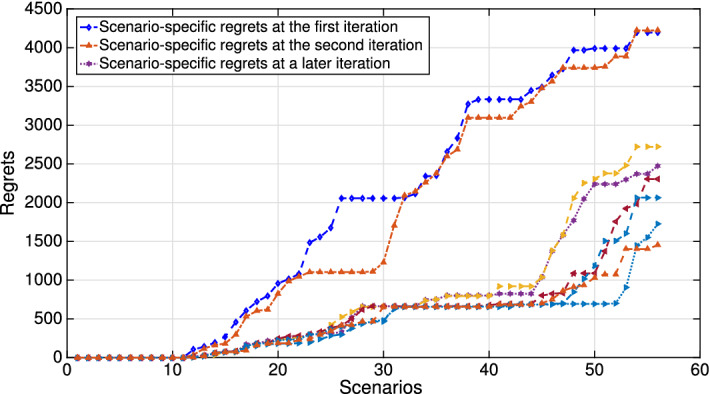
Regrets at different iterations of Benders decomposition procedure (Algorithm 1)

In Fig. 15b, the changes in regret are multiplied by the corresponding scenario probabilities. We observe that the mitigation is likely to reduce the regrets corresponding to the disruptions of middle length. This happens at a cost of shorter disruptions (i.e., which are typically low-cost). This is in line with the rationale for the mitigation, which suggests spending more in good times in order not to be affected should a significant disruption occur. Lastly, Fig. 16 shows the reduction in scenario-specific regrets following multiple iterations of Algorithm 1.

**Fig. 17:**
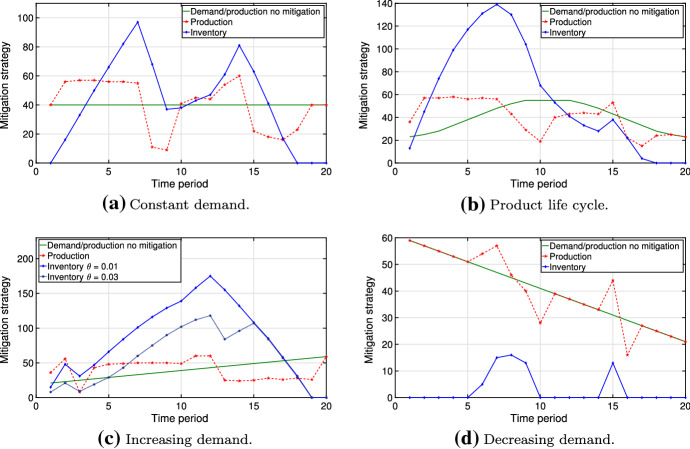
Optimal mitigation strategies for different demand paths with $\alpha =0.05$, $\beta =0.5$, $\theta =0.01$, $\gamma =2500$

A natural extension to our work would be a model with stochastic demand, which would require an additional chance constraint or an expectation minimization. Although accounting for this type of uncertainty is beyond the scope of this article, we test different shapes of demand patterns, including constant, increasing, decreasing and a product life cycle curve (bell shape). In Fig. 17, we observe that the optimal production drops below the demand curve for every pattern, while the decision-maker mitigates disruptions by creating a peak in mitigation inventory. The most pronounced (least pronounced) peak in mitigation inventory is observed when there is an increasing (decreasing) demand pattern. Similar to Fig. 14 for increasing loss threshold $\gamma$, Fig. 17c demonstrates the risk clustering effect for increasing risk tolerance $\theta$, suggesting inventory aggregation in some regions of the horizon as more disruption scenarios can be selected into the risk budget.

## Conclusion and outlook

The risk of supply chain disruptions prompts decision-makers to construct risk mitigation strategies to prepare for unforeseen events. In this article, we developed an optimization model to minimize total production costs for a supply chain subject to uncertain disruptions. We formulated a convex network flow problem under a chance constraint bounding “regrets” in disrupted scenarios. The loss after a disruption was measured as a distance between two optimization problems (reactive and anticipative), whose solution depends on the risk mitigation strategy.

We took a relatively general approach by assuming convex but not necessarily differentiable cost functions: This gives decision-makers more flexibility in modeling the cost structure and allows them to account for production switching effects, multiple production facilities or the use of contract manufacturers with different costs. We can model both full production disruptions and disruptions that imply a change from one convex cost function to another. Moreover, although our model assumes a finite time horizon, it can be applied in the context of a rolling horizon planning. That is, a decision-maker would only implement the first *m* periods of a suggested resilient plan, where *m* is determined by the willingness of the decision-maker to pay for re-planning. An additional benefit of such an approach is that it can sidestep undesired or erratic behavior induced by leftover inventory or backlog at the end of the planning horizon. Note however that in order for the two-stage structure of the model resulting from the introduction of our concept of regret to make sense re-planning needs to be an action a decision-maker does not want to undertake often, limiting the types of products for which our model is applicable. Specifically, our model is a better fit for products subject to a limited life cycle. Such life cycles often decompose into three demand phases: An initial phase of increasing demand, a second phase during which demand is stationary, and a final phase in which demand decreases. Although the numerical results we presented assumed stationary demand, this is not a modeling necessity, and therefore our model can be used to study all three phases.

Much of our numerical analysis focused on understanding how the resilient plan behaves depending on the properties of disruption scenarios. Specifically, we determined which disruption scenarios are likely to enter the risk budget and which tend to be explicitly mitigated against. Our model is applicable for a wide range of disruptions and allows decision-makers to optimize their risk mitigation strategy by evaluating trade-offs between holding inventory or leveraging agility capacity under different demand patterns. Under no mitigation, the most costly disruption scenarios would be those starting in the middle of the planning horizon and persisting until the end. Implementation of a risk reduction strategy helps decision-makers to decrease these costs, increasing the inventory levels available beforehand. The largest increase in mitigation inventory is necessary when disruptions occur early and are not resolved quickly or when disruptions occur late but do not allow for reaction time. Importantly, in our optimal resilient plan, the regrets decrease for all types of disruptions except very short ones, including those that are very unlikely and are thus selected into the risk budget. Furthermore, the availability of agility capacity, i.e., of partial production during a disruption, reduces the expected regret even if its cost is higher than the production cost during undisrupted periods. Finally, we observed and characterize a risk clustering behavior which, to our knowledge, has not been previously addressed in the literature and will require future research.

Another natural extension of our research includes a model with stochastic demand. This generalization requires additional chance constraints or an expectation minimization. For this, it would be necessary to account for the structure of Benders subproblems to speed up the algorithm and to efficiently determine the optimal solution. Future research might also focus on analyzing multi-stage models accounting for multiple disruptions within a given planning horizon.
